# Do Relapses Follow ANCA Rises? A Systematic Review and Meta-Analysis on the Value of Serial ANCA Level Evaluation

**DOI:** 10.3389/fmed.2022.844112

**Published:** 2022-07-04

**Authors:** Aram Al-Soudi, Yosta Vegting, Paul L. Klarenbeek, Marc L. Hilhorst

**Affiliations:** ^1^Department of Rheumatology, Amsterdam UMC, University of Amsterdam, Amsterdam, Netherlands; ^2^Rheumatology and Immunology Center (ARC), Amsterdam UMC, University of Amsterdam, Amsterdam, Netherlands; ^3^Department of Internal Medicine, Section of Nephrology, Amsterdam UMC, University of Amsterdam, Amsterdam, Netherlands; ^4^Department of Rheumatology, Spaarne Gasthuis, Hoofddorp, Netherlands

**Keywords:** ANCA-associated vasculitis (AAV), anti-neutrophil cytoplasmic antibodies (ANCA), biomarker (BM), relapse, flare

## Abstract

**Objectives:**

ANCA-vasculitis (AAV) patients frequently suffer from relapses and risk subsequent organ damage. There is much debate on the value of serial ANCA level evaluation to monitor disease activity. We aimed to evaluate the association between ANCA rises and disease relapses at (I) moment of the rise, (II) within 6 months or (III) within a year from the rise.

**Methods:**

3 databases (MEDLINE, EMBASE, COCHRANE) were searched from 1993 through September 2021. We included studies that reported relapse incidence within 12 months after an ANCA rise measured by antigen-specific immunoassays in peripheral blood of AAV patients in remission. Quality assessment was performed using QUADAS-2. Finally, a meta-analysis was carried out to estimate average OR using a random effects model.

**Results:**

Twenty unique studies were included. The methodological quality was limited due to risk of selection bias. An ANCA rise often preceded a disease relapse within 6 months (OR 3.65, 95% CI 1.66–8.03) and less often within 12 months (OR 2.88, 95% CI 1.21–6.88), while it was not indicative of a concurrent relapse (OR 0.13, 95% CI 0.03–0.53). Once a relapse is diagnosed, ANCA is significantly more often present than not (OR 10.80, 95% CI 3.82–30.55). As expected based on clinical, technical and methodological variability between studies, there was substantial heterogeneity across studies in all analyses (I2 = 70–87%).

**Conclusion:**

In previously ANCA-positive patients, the ANCA test is often positive upon clinical suspicion of a disease relapse. Patients with a rise in ANCA are at risk of encountering disease relapses in the upcoming 6 or 12 months.

## Graphical Abstract

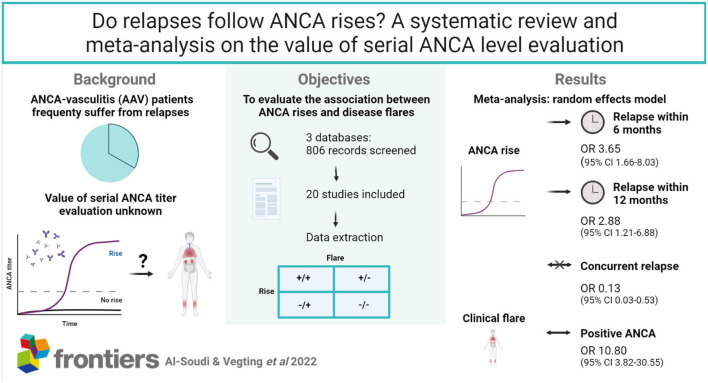

## Key Messages

1. ANCA rises often precede disease relapses in the upcoming 6 or 12 months.
2. In previously ANCA-positive patients, the ANCA test is often positive upon clinical suspicion of a disease relapse.

## Introduction

ANCA-associated vasculitis (AAV) refers to a group of vasculitides associated with the presence of antineutrophil cytoplasmic antibodies (ANCA). AAV comprises the clinical diagnoses of granulomatosis with polyangiitis (GPA), microscopic polyangiitis (MPA) and eosinophilic granulomatosis with polyangiitis (EGPA). Although survival has improved over the past decades, mortality remains significantly increased, especially in patients with renal involvement (1, 2). Optimal treatment of AAV is challenging as the disease course is unpredictable. Some patients will experience relapses (after cessation of therapy) while others will not. Relapses induce and accelerate further organ damage, which is shown by the association between renal relapses and the incidence of end-stage kidney disease (ESRD) (3). Therefore, there is a pressing need for accurate biomarkers to monitor and predict disease activity in patients (4, 5). ANCA target predominantly proteinase-3 (PR3) and myeloperoxidase (MPO), both cytoplasmic components of neutrophils and monocytes. ANCA are being used extensively in diagnosing AAV, and are usually measured using immunoassays such as enzyme linked immunosorbent assays (ELISA) selectively measuring PR3- or MPO- antibodies. Although the place of ANCA testing in the diagnostic workup of AAV is undisputed, heterogeneous results have been obtained when investigating the predictive value of ANCA rises (6, 7). This heterogeneity is the result of clinical, technical and methodological factors that influence the correlation between ANCA rises and relapses. The disease severity of included patients, presence of renal involvement and persistence of ANCA positivity differs between cohorts (8). Technical and methodological factors comprise differences in sampling intervals, the use of both indirect immunofluorescence (IIF) and ELISA methods, and unclear chronological relations between ANCA changes and relapses. As a result, it remains unclear what ANCA rises mean when measured during remission, whether they precede subsequent relapses and/or if disease relapses are associated with positive ANCA levels.

Over the years the use of ELISA to primarily detect ANCA has increased and has resulted in an international consensus to use immunoassays as primary screening method, without the categorical need for IIF (9). A systematic review supported this notion and determined that especially the sensitivity of ANCA detection considerably increases with the use of immunoassays (10). The question whether an increase in ANCA levels leads to an increase in disease activity within a clinically relevant timeframe (e.g. within 6 months) remains open.

This systematic review provides a comprehensive literature search, followed by quality assessments and meta-analysis to explore whether ANCA level increases as measured by antigen-specific immunoassays associate with disease relapses at moment of the rise, within 6 months of the rise or within a year from the rise.

## Methods

### Literature Search Strategy

For this systematic review and meta-analysis, MEDLINE, EMBASE and COCHRANE were searched by two investigators (AA, MH) for 1) articles on ANCA measurements predicting relapse and 2) diagnosing disease activity in ANCA associated vasculitis (AAV). No restrictions were selected for publication date (inception to September 2021) and/or language. Articles were filtered on studies performed in humans. A combination of MeSH terms (Antibodies, Antineutrophil Cytoplasmic, Recurrence) and title + abstract (tiab) terms (ANCA, relapse, biomarker, disease activity) was used to identify relevant articles (Supplementary Table S1). Cross-references from reference lists of found studies and similar articles from PubMed were reviewed as well.

### Study Selection

**Figure 1:**
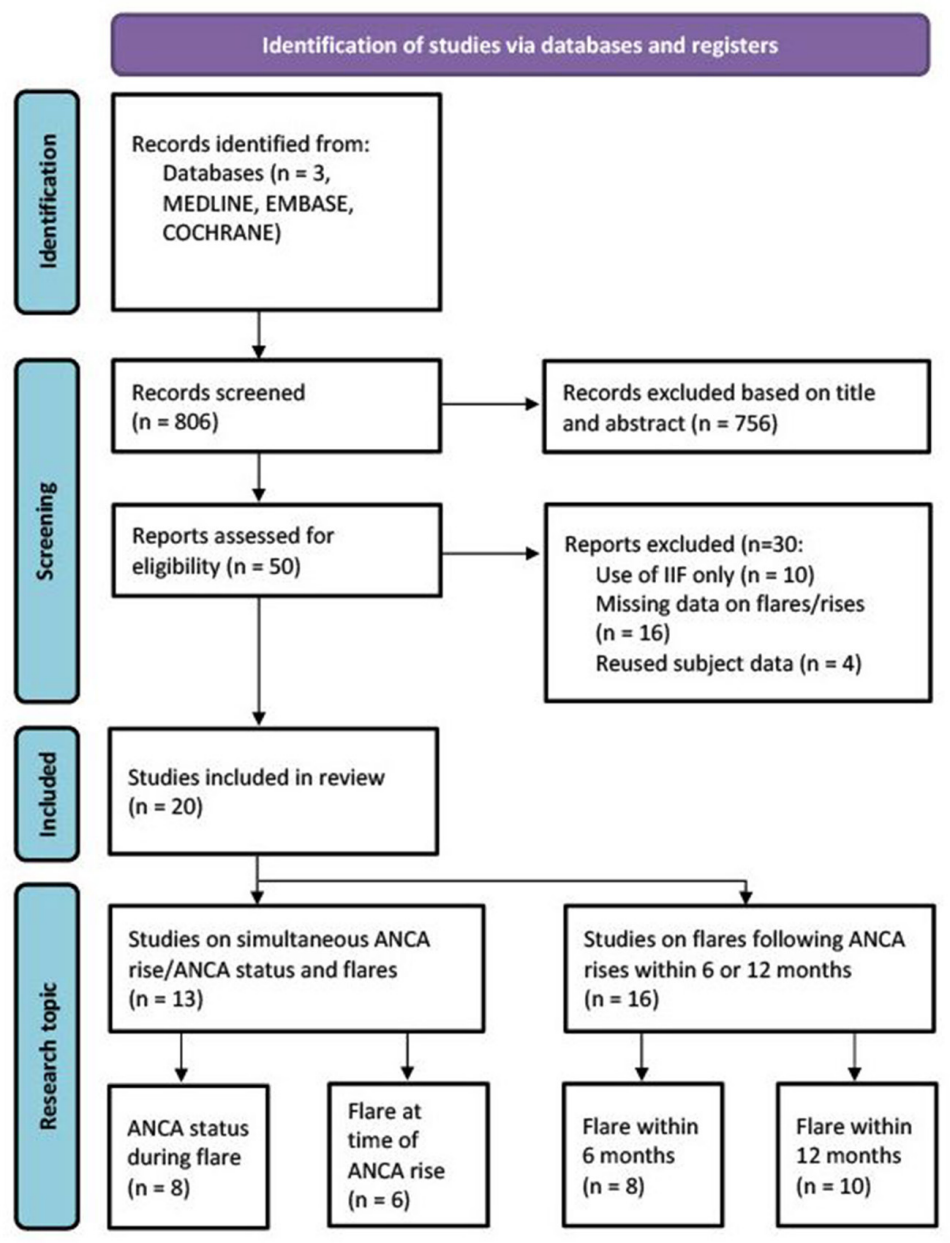
Schematic overview of study selection procedure (12).

Found articles (last search on 01-09-2021) were screened on title and abstract by AA, MH and PLK independently. After title and abstract screening, selected studies were reviewed for final inclusion or exclusion (Figure 1). Inclusion criteria consisted of the use of antigen-specific immunoassays, a timeframe from measurement to relapse of a maximum of 12 months and a definition for a rise as either negative to positive conversion or a ANCA level increase. When patient cohort(s) were used in multiple studies, only one study was included. Since the majority of studies are performed using ELISA, we selected ELISA studies over alternative solid-phase immunoassays (such as FEIA) to increase the comparability between studies. No disagreements on study inclusion were found. In case of missing crucial data elements to calculate sensitivity, specificity and subsequent measures such as odds ratios, the study was excluded.

### Data Collection

Data extraction was performed by AA and YV independently. Name of first author, year of publication, study type, number of included patients, percentage of renal involvement, used immunoassay, definition of ANCA rise, and sampling interval were extracted. The slope of ANCA increase (%/month) was calculated by dividing the percentage of ANCA increase by the longest sampling interval. Studies were further separated on type of ANCA (PR3, MPO or pooled), time to relapse (rise during relapse, 1–6 months or 6–12 months), and the definition of a rise. From several studies we obtained data regarding ANCA rises during a relapse and prior to a relapse. From all selected studies the total number of relapses and total number of patients were extracted and four groups were created (rise+relapse+, rise-relapse+, rise+relapse-, rise-relapse-).

### Quality Assessment

All included studies were assessed independently by AA and YV using the Quality Assessment Tool for Diagnostic Accuracy (QUADAS-2), in accordance with systematic review recommendations (11). According to QUADAS-2 guidelines, the content of the tool was first tailored to the two main research questions; to investigate the simultaneous presence of ANCA (rises) and relapses, and to investigate relapses that would follow ANCA rises within 6 or 12 months. This was followed by an independent pilot to reach agreement on the rating method by YV and MH. The QUADAS-2 supports a critical quality assessment of studies by providing four domains to assess: patient selection, index test, reference standard and timing; with subsequent questions in each domain. A study is regarded as having a high potential risk of bias when 2 or more negative points are found in each section. In case of discordance between quality assessments of AA and YV, the study was discussed with the other authors.

### Statistical Analysis

To study ANCA positivity or rise during a relapse, odds ratios were calculated by dividing the odds of having a clinical flare accompanied by a positive or rising ANCA test, divided by the odds of having a clinical flare accompanied by a negative or stable ANCA test. To study relapses following ANCA rises, odds ratios were calculated by dividing the odds of having an ANCA rise in 6 or 12 months before a clinical flare is diagnosed, divided by the odds of having an ANCA rise without the presence of a clinical flare in the following months. Finally, a meta-analysis based on the odds ratio was performed to estimate the odds of having a relapse when ANCA rises as opposed to a non-ANCA-rise. A random effects model was used and 95% confidence intervals (CI) were calculated. Four meta-analyses were performed: ANCA positivity during a relapse, having a relapse when ANCA rises, having a relapse within 6 months of an ANCA rise and having a relapse within 12 months of an ANCA rise. Statistical heterogeneity was assessed by Chi-squared test (Chi^2^) and between-study inconsistency was quantified by the I^2^ statistic. In subsequent analyses, studies were pooled based on whether they were PR3-ANCA-only, MPO-ANCA-only or pooled ANCA. Forest plots were generated using Review Manager 5.4 software.

## Results

### Final Study Selection and Characteristics

The literature search provided 806 results. These included all articles found in the last known meta-analysis on this subject from 2012(6). After title and abstract screening, 50 studies were selected for full-text review (Figure 1) (12). Inclusion and exclusion criteria were reviewed and 30 studies were excluded due to the use of IIF only (*N* = 10), missing information to complete calculations or to subdivide studies in 6 or 12 month prediction analyses (*N* = 16) or due to reuse of patient cohorts (*N* = 4) (Supplementary Table S2). 20 studies were included (Table 1). In total, 13 studies were used for simultaneous ANCA rise and disease relapse analysis, with eight studies investigating whether ANCA is positive during disease relapse and six studies investigating whether a relapse is simultaneously present with an ANCA rise. A total of 16 studies were used for the meta-analyses of ANCA rises preceding subsequent relapses within 6 or 12 months. Some studies provided information on both simultaneous ANCA rise and disease relapse, as well as prediction of disease relapses. Selected studies were published between 1993 and 2019. Nine of 20 studies were prospectively conducted. Detailed characteristics of included studies are provided (Table 1).

**Table 1:** Overview of included studies.

								**ANCA Rise**			
**Study**	**Type**	**Pat. (n)**	**Renal involv (%)**	**Immuno assay**	**Coating**	**Dil**.	**Anca type**	**Level↑ (%)**	**Slope (%/m)**	**-/+ Convers**.	**Interval (m)**
Ara et al. (13)	Cohort (→)	25	100	D-ELISA	Hn	1/100	MPO	NA	NA	Yes	3
Boomsma et al. (14)	Cohort (→)	100	NA	D-ELISA	Hn	PR3: 1/100 1/300 MPO: 1/60 1/180	Both*	175 (ROC)	87.5	NA	2
Damoiseaux et al. (15)	Case-control (←)	46	NA	C-ELISA	Hn	1/100	PR3	200 (ROC)	66.7	NA	3
De'Oliveira et al. (16)	Cohort (←)	56	NA	D-ELISA	Hn	NS	Comb.	120	40	Yes	2–3
Dolman et al. (17)	Case-control (←)	8	NA	C-ELISA	Hn	1/100	PR3	200	200	NA	1
Finkielman et al. (18)	Cohort (→)	101	54	C-ELISA	NS	NS	PR3	200	33.3	NA	2–6
Fussner et al. (19)	RCT (→)	131	65	C-ELISA	NS	NS	PR3	200	33.3	Yes	0.25–6
Han et al. (20)	Cohort (←)	48	NA	D-ELISA	NS	NS	Comb.	400	133.3	NA	2–3
Jayne et al. (21)	Cohort (→)	60	60	D-ELISA	Hn	1/50	Comb.	130	130	Yes	1
Jones et al. (22)	RCT (→)	29	100	ELISA	NS	NS	Comb.	NA	NA	Yes	6
Kemna et al. (8))	Cohort (←)	166	63	D-ELISA + FEIA	Hn	1/50	Comb.	233 (ROC)	78	Yes	3
Lurati-Ruiz et al. (23)	Cohort (←)	36	72	ELISA	NS	NS	Comb.	200	NA	NA	NA
McClure et al. (24)	Cohort (←)	57	37	C-ELISA	NS	NS	PR3	200	33.33	Yes	3–6
Miloslavsky et al. (25)	RCT (→)	170	66	D-ELISA	Hn and Hr	1/100	Comb.	200	100	Yes	0.25–2
Nowack et al. (26)	Cohort (←)	18	78	C-ELISA	Hn	1/100	PR3	150	100	NA	0.5–1.5
Segelmark et al. (27)	Case-control (←)	14	70	C-ELISA	Hn	1/80	PR3	150	NA	NA	Not serial
Specks et al. (25)	RCT (→)	197	66	D-ELISA	Hn and Hr	1/100	Comb.	200	66.7	Yes	0.25–3
Terrier et al. (28)	Cohort (←)	38	50	ELISA	NS	1/20	MPO	NA	NA	Yes	NA
Watanabe et al. (29)	Cohort (→)	195	78	FEIA, CLIA, ELISAs	NS	NS	MPO	NA	NA	Yes	3–6
Yamaguchi et al. (30)	Cohort (→)	118	100	ELISA	Hn	1/500 or 1/101	MPO	200	200	Yes	1

→ , prospective; ←,retrospective; Pat., number of patients included in analysis; involv., involvement; * = only data on PR3 at 6 months; NS, not specified; NA, not annotated in study; Simult. Relapse, relapse and ANCA measurement done at the same time; 6 m, relapse follows an ANCA rise within 6 months; 12m, Relapse follows an ANCA rise within 12 months; D, direct ELISA; C, Capture ELISA; FEIA, fluorescent-enzyme immuno-assay; CLIA, Chemiluminescence immunoassay; Hn, human native; Hr, human recombinant. Dil., dilution; Both, PR3 and MPO are investigated separately in study; Comb., Combined, no distinction is made between ANCA subtype in study; Level ↑, ANCA level increase in %; -/+ Convers., negative to positive conversion; ROC, ANCA rise is defined using a slope of ANCA level change and subsequent ROC analyses; m, month; Interval, sampling interval.

### Quality Assessment of Selected Studies

A risk of bias inventory was made and schematically overviewed for each of the conditions (Supplementary Tables S3–5). Reasons for scoring risk of bias are annotated (Supplementary Tables S6, S7).

### What Is the ANCA Status When a Relapse Is Diagnosed?

**Figure 2:**
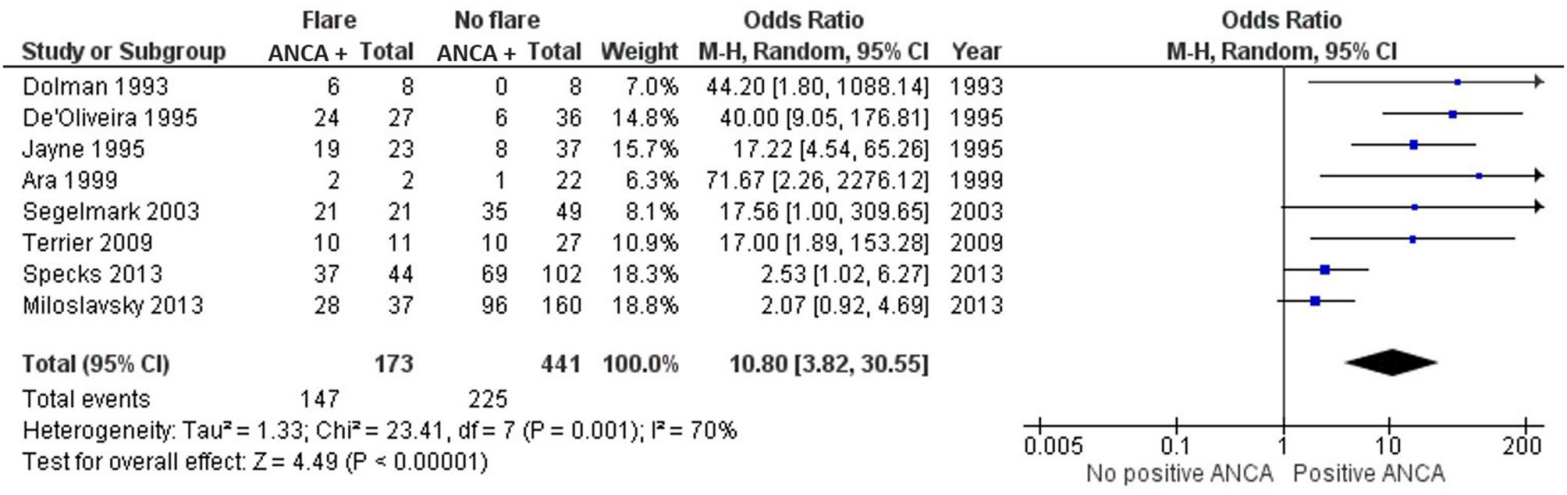
Meta-analysis summarizing the data regarding ANCA positivity once a relapse is diagnosed. Odds ratio with 95% confidence interval is displayed in the forest plot.

Eight studies investigated the moment of relapse and tested for the positivity of ANCA after patients had been in remission prior (13, 16, 17, 21, 25, 28, 31, 32). None of these studies had included individuals with newly diagnosed AAV. Only one of the seven studies, Miloslavsky et al. (25), demonstrated an insignificant OR of 2.07 (CI 0.92–4.69) (25). All other studies favor the presence of a positive ANCA when a relapse is present, with a grand total OR of 10.80 (3.82–30.55) (Figure 2). Dolman et al. (17) and Segelmark et al. (31) included only PR3-ANCA positive individuals, whereas Ara et al. (13) and Terrier et al. (28) only included MPO-ANCA positive individuals. Ara et al. (13) and Dolman et al. (17) demonstrated the highest OR with 71.67 (2.26–2276.12) and 44.20 (1.80–1088.14), respectively, but had the lowest number of patients. The results significantly favor the detectability of ANCA at the moment of disease relapse (Figure 2).

### What Does a Rise in ANCA Indicate?

**Figure 3:**
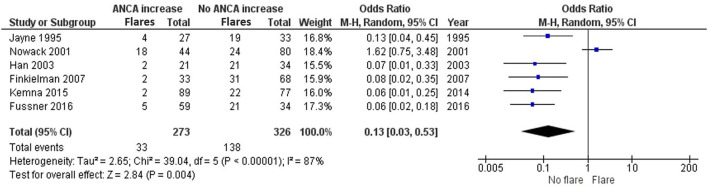
Meta-analysis summarizing the data regarding having a relapse when ANCA increases. Odds ratio with 95% confidence interval is displayed in the forest plot.

ANCA is often used to monitor AAV patients, even though fluctuations are common during follow-up. We reviewed the literature to understand whether a concurrent ANCA rise indicates a disease relapse or might associate with disease relapses in the future. Six studies investigated whether an ANCA rise is simultaneously associated with a disease relapse (8, 18–21, 26). In these studies, an ANCA rise was defined as 1.3–4-fold elevation in ANCA level. Jayne et al. (21), Han et al. (20), Finkielman et al. (18), Kemna et al. (8), and Fussner et al. (19) strongly favor an absence of a disease relapse, as opposed to the presence of a disease relapse, simultaneously with an ANCA increase. Nowack et al. (26) slightly trends toward favoring a relapse instead, with an OR of 1.62 (0.75–3.48). Overall there is an OR of 0.13 (0.03–0.53) that significantly favors not having a relapse, as opposed to the presence of a relapse, simultaneously with an ANCA increase (Figure 3).

### Do ANCA Increases Precede Future Disease Relapses?

**Figure 4:**
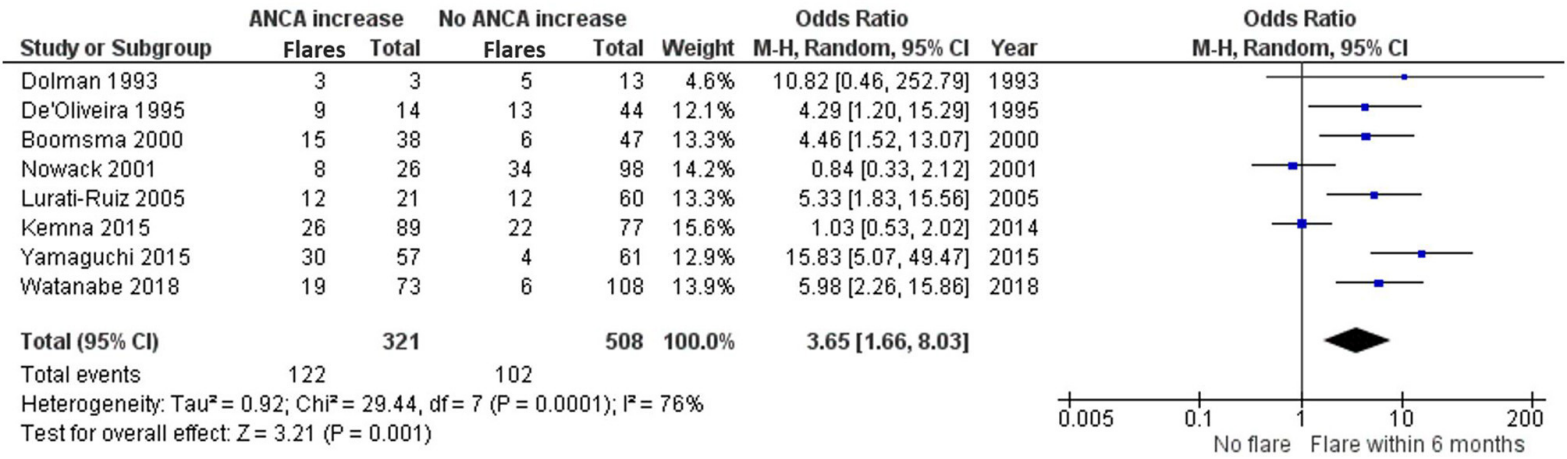
Meta-analysis summarizing the data regarding having a relapse within 6 months of an ANCA increase. Odds ratio with 95% confidence interval is displayed in the forest plot.

ANCA increases could be indicative of an ongoing or starting inflammatory process that could indicate future relapses. We set out to investigate, within clinically relevant time frames, if this was the case. Eight studies investigating ANCA rises with subsequent relapse within 6 months of the rise are included in the meta-analysis (8, 14, 16, 17, 23, 26, 29, 30) (Figure 4).

De'Oliveira et al. (16), Boomsma et al. (14), Lurati-Ruiz et al. (23), Yamaguchi et al. (30) and Watanabe et al. (29) all significantly favor a relapse within 6 months, as opposed to no relapse, after a measured ANCA increase. Additionally, the study by Dolman et al. (17) trends toward a relapse within 6 months with an OR of 10.82 (0.46–252.79). The studies by Nowack et al. (26) and Kemna et al. (8) remain inconclusive. The grand total OR of having a relapse within 6 months of an ANCA increase compared with not having a relapse is 3.65 (1.66–8.03).

**Figure 5:**
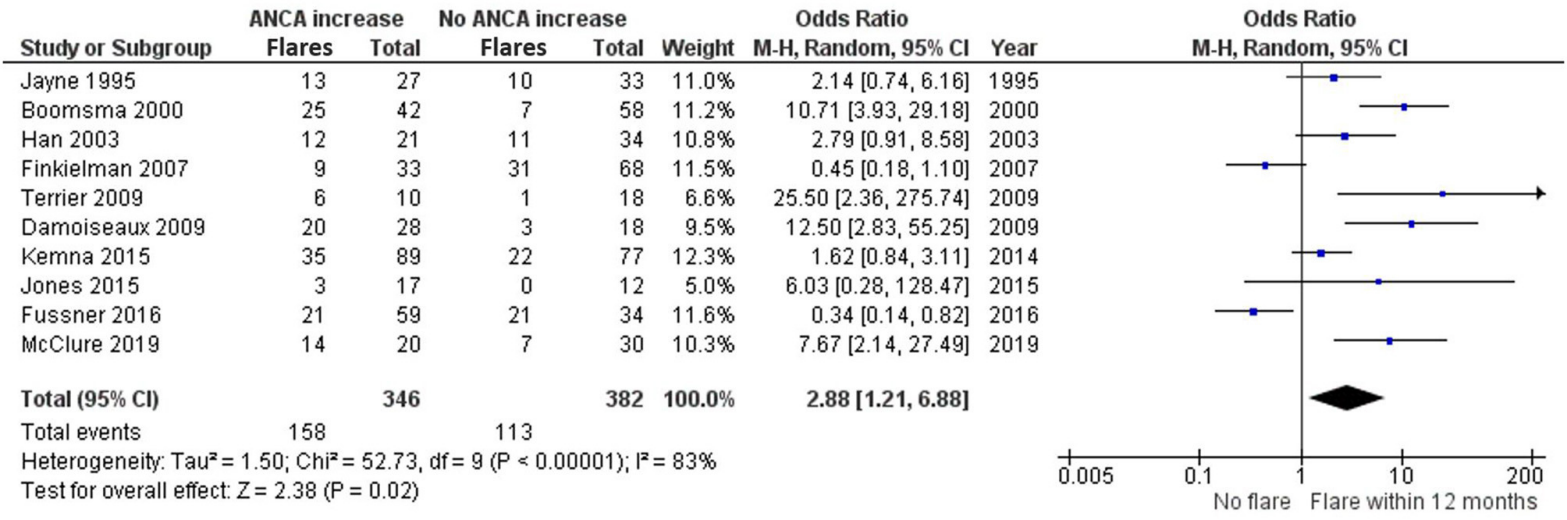
Meta-analysis summarizing the data regarding having a relapse within 12 months of an ANCA increase. Odds ratio with 95% confidence interval is displayed in the forest plot.

Ten studies investigated ANCA rises with subsequent relapses within 12 months of the rise (8, 14, 15, 18–22, 24, 28) (Figure 5). Part of these studies overlap with the 6 months analysis, but have looked separately at relapses within 12 months. Boomsma et al. (14), Terrier et al. (28), Damoiseaux et al. (15), and McClure et al. (24) significantly favor the presence of a relapse as opposed to no relapse, within 12 months of an ANCA increase. Additionally, Jayne et al. (21), Han et al. (20), Kemna et al. (8), and Jones et al. (22) trend toward favoring a relapse within 12 months of an ANCA increase. However, Finkielman et al. (18) and Fussner et al. (19) favor no relapse instead, making the 12-month data slightly more variable compared with the 6-month data. This also resulted in a slightly lower, yet significant grand total OR of 2.88 (1.21–6.88), favoring a relapse as opposed to no relapse within 12 months of an ANCA increase.

### Differences Between PR3-ANCA and MPO-ANCA Positive Patients

In previous analyses PR3-ANCA and MPO-ANCA patients were pooled. To extrapolate our findings to patient groups, it would be of interest to understand whether disease relapses are predicted more accurately by changes in one of the two ANCA types. Within 6 months of a ANCA rise, Yamaguchi et al. (30) and Watanabe et al. (29) investigated MPO-ANCA only. A grand total OR of 9.29 (3.59–24.07) was reached in favor of having a relapse, as opposed to no relapse, within 6 months after MPO-ANCA increase (Supplementary Figure S1). In contrast, Dolman et al. (17), Boomsma et al. (14) and Nowack et al. (26), all investigating PR3-ANCA positive patients, only showed a trend toward favoring having a relapse within 6 months of a PR3-ANCA increase at an OR of 2.46 (0.57–10.62) (Supplementary Figure S2).

At 12 months, Boomsma et al. (14) and Terrier et al. (28) both favor a relapse within 12 months of an MPO-ANCA increase with an OR of 27.11 (4.14–177.41) (14, 28) (Supplementary Figure S3). However, both studies have studied a low number of patients. For PR3-ANCA positive patients only, the results are heterogeneous. Finkielman et al. (18) and Fussner et al. (19) favor no relapse within 12 months of a PR3-ANCA increase with OR of 0.45 (0.18–1.10) and 0.34 (0.14–0.82) respectively. However, Boomsma et al. (14), Damoiseaux et al. (15) and McClure et al. (24) significantly favor a relapse within 12 months of a PR3-ANCA increase, shifting the grand total of the five studies toward favoring a relapse within 12 months at an OR of 2.53 (0.52–12.44) (Supplementary Figure S4). Although the results are widespread, clinical characteristics of the five studies are similar for ANCA increase definition, but the two studies that trend toward no association are prospective cohort studies (18, 19) (Table 1).

## Discussion

The use of serial serum ANCA level evaluation during remission is questionable and varies in daily clinical practice. In this systematic review and meta-analysis we demonstrate that during follow-up of AAV patients who are in remission, an ANCA rise often preceded a disease relapse within 6 months and to a lower extent within 12 months, while an increase in ANCA level is not indicative of an ongoing relapse. Once a relapse is diagnosed, ANCA is significantly more often present than not. In further follow-up of patients, MPO-ANCA increases were more significantly associated with future disease relapses than PR3-ANCA increases.

The complexity of ANCA level evaluation has frequently been discussed and is influenced by many factors, such as disease severity, follow-up, treatment and variation in ANCA test methods (8). Whether the serological subtype affects the association between ANCA rises and relapses remains unclear since ANCA response is heavily influenced by induction regimens (19). While data from our meta-analysis suggests that there was a strong association between ANCA rises and relapses in MPO-ANCA positive patients, a recent cohort study of Van Dam et al., (33) did not find an association with reappearance of MPO-ANCA, but only of PR3-ANCA. All studies on MPO-ANCA included in our meta-analysis used cyclophosphamide for remission-induction therapy, while Rituximab was used in the latter study. These results indicate differences in the role of MPO- and PR3-ANCA during disease reactivation (34) and treatment-specific interactions, but the exact mechanisms remain to be elucidated.

The variable association between ANCA and relapses could also be explained by modulation of immune responses on a tissue level. While it is known that ANCA activate the innate immune system leading to necroinflammation and endothelial cell damage (35, 36), disease manifestations are not always present in patients with elevated ANCA levels. Therefore, we speculate that the autoimmune cascade induced by ANCA is counterbalanced by mechanisms that attempt to maintain homeostasis. For example, serum anti-inflammatory cytokine IL-10 levels are increased in ANCA patients (27, 37, 38) and inhibit inflammation caused by neutrophils (39). In many cases, ANCA level fluctuations do not lead to substantial organ damage and no relapse is following. However, when these feedback mechanisms are overwhelmed and local damage is increasing, complaints develop and a relapse is diagnosed. Therefore, ANCA rises and relapses are not exhibited at the same time, but are clearly linked.

Results of this meta-analysis extend our knowledge of the utility of ANCA level evaluation and are consistent with a previous systematic review (6), demonstrating that ANCA rises have a significant association with future disease relapses. Key strengths are first, that studies are selected for the use of a similar antigen-specific immunoassay, which minimizes bias introduced by comparing multiple testing techniques. It has to be noted that no recommendations exist on what assay is preferred for monitoring or follow-up of AAV patients (9). Second, relapses are categorized according to clinically relevant timeframes from ANCA rise to relapse. And third, the PR3-ANCA and MPO-ANCA subtypes are separately studied in sub-analyses. Therefore, the combination of clinically relevant inclusion criteria combined with an extensive quality assessment provides clinicians with information on the value of ANCA testing during a relapse and the association with future disease relapses.

Yet, weaknesses in our systematic review may have also arisen from the choice to include all types of study designs. The patients, methods and techniques used in the included studies were diverse, leading to statistical heterogeneity. The study of Kemna et al. (8) identified multiple factors that influence the association between ANCA rises and relapses, such as renal involvement, persistent ANCA positivity, slope, and sampling interval. As can be seen from Table 1, these factors differed between the included patients cohorts, likely affecting outcomes. Also, over half of the studies showed a high risk of bias by quality assessment (Supplementary Tables S6, S7), which can lead to under- or overestimation of the observed effect. For example, case-control studies may lead to overestimation of the diagnostic accuracy (40), the majority of studies did not specify which patients remained ANCA-positive in remission, therefore, inclusion of these patients could affect the results on ANCA status during a relapse. Furthermore, ANCA rises and time intervals were frequently not pre-defined, nor blinding for the ANCA test was performed. Since individual patient data was rarely available, we could not adjust for potential confounders or look into patient-specific effects, therefore, we provided estimates of the odds ratios for the total group of ANCA patients.

What do our results mean for clinical practice? In case of a disease relapse the ANCA is likely to be positive, similar to the situation at time of AAV diagnosis. Therefore, in case of a negative ANCA test, alternative diagnoses should logically be considered. Second, an ANCA rise is not necessarily related to a concurrent relapse, but patients are more likely to experience a disease relapse in the 6 months following an ANCA increase. These results provide further support to monitor patients with an ANCA rise more closely and raise the question if patients should be treated pre-emptively to prevent a potential disease relapse with subsequent organ damage. On the other hand, over half of the patients with an ANCA rise do not experience a relapse within a year, and these patients risk complications of overtreatment such as infections. Starting or escalating immunosuppressive treatment could be beneficial for high-risk patients. This is confirmed in a small randomized trial which showed that there were significantly fewer major relapses in the group that was pre-emptively treated with high dose cyclophosphamide and prednisolone after an ANCA rise (41). In addition, two retrospective cohorts found a significant reduction in the incidence of relapses in patients in whom maintenance therapy was intensified (20, 30). These studies are limited by small sample sizes and differences in treatment options. Based on our findings and these promising results, we believe pre-emptive treatment will form the future clinical practice guided by prediction models including specific patient characteristics (severity of disease, renal involvement, persistence of ANCA, type of ANCA, glycosylation profiles), environmental characteristics (seasonal influences) and testing characteristics (immunoassay used, sampling interval) to minimize potential side effects of overtreatment and maximize efficacy in selecting individuals at high risk of developing a vasculitis flare (42–44). Further research will investigate potential applicability, benefits and cost-effectiveness of changing this clinical practice and tailoring therapy for specific patient groups.

## Conclusion

Although an AAV relapse is associated with positive ANCA, rises in ANCA level are not indicative of a concurrent disease relapse. However, ANCA increases do associate significantly with higher odds of having a disease relapse within the first 6 and 12 months after measurement. This association is strongest in MPO-ANCA positive patients and loses significance in PR3-ANCA positive patients. Our meta-analysis confirms that in previously ANCA-positive patients, the ANCA test is often positive upon clinical suspicion of a disease relapse and an increasing ANCA may be helpful to identify patients that are more at risk of encountering disease relapses in the upcoming 6 or 12 months, and for whom pre-emptive treatment could be a realistic possibility.

## Author Contributions

AA and MH set up the literature search strategy and performed the search. Study selection was performed by AA, MH, and PK. The quality of studies was assessed, data extraction, and data analysis was performed by AA and YV. All authors contributed in writing the manuscript and approved the final manuscript.

## Funding

This work was supported by the Dutch Kidney Foundation [Grant number 19OK007 to MH and YV] and ZonMW [VENI grant number 91617058 to PK].

## Conflict of Interest

The authors declare that the research was conducted in the absence of any commercial or financial relationships that could be construed as a potential conflict of interest.

## Publisher's Note

All claims expressed in this article are solely those of the authors and do not necessarily represent those of their affiliated organizations, or those of the publisher, the editors and the reviewers. Any product that may be evaluated in this article, or claim that may be made by its manufacturer, is not guaranteed or endorsed by the publisher.
