# Supplementary material for: Do Relapses Follow ANCA Rises? A Systematic Review and Meta-Analysis on the Value of Serial ANCA Level Evaluation
Source: Front Med (Lausanne). 2022 Jul 4;9:844112. doi: 10.3389/fmed.2022.844112 (PMC9289208; doi:10.3389/fmed.2022.844112)
Supplement: Supplementary file 1 [file Data_Sheet_1.pdf]

Supplementary Material

Search command		Search command		Search command	
"Antibodies, Antineutrophil Cytoplasmic"[Mesh]	OR	"anca"[tiab]	OR	"ANCA"[tiab]	*AND*
"Vasculitis"[Mesh]	OR	"Anti-Neutrophil Cytoplasmic Antibody-Associated Vasculitis"[Mesh]			*AND*
"Recurrence"[Mesh]	OR	"relapse"[tiab]	OR	"flare"[tiab])	

Supplementary Table S1. Full search strategy. Each row represent a search command between brackets, coupled by 'AND'.

Reason for exclusion	Studies (n=30)	Year
Use of IIF only		
	Cohen Tervaert	1989
	Cohen Tervaert	1990
	Gaskin	1991
	Petterson	1992
	Gordon	1993
	Kerr	1993
	Davenport	1995
	Girard	2001
	Keogh	2003
	Land	2017
Missing information on numbers and/or relapses		
	Nolle	1989
	Halma	1990
	Power	1995
	Blockmans	1999
	Kyndt	1999
	Gisslen	2002
	Sanders	2006
	Lin	2009
	Kalsch	2010
	Rasmussen	2013
	Thai	2013
	Moog	2014
	Draibe	2015
	Domingues	2015
	Verstockt	2015
	Tanaka	2020
Reused subject data		
	Boomsma	2003
	Kemna	2017
	Thompson	2020
	Damoiseaux	2005

Supplementary table S2. Overview of excluded studies per reason of exclusion

Study	Risk of bias				Applicability concerns		
	Patient selection	Index test	Reference standard	Flow and timing	Patient selection	Index test	Reference standard
Dolman 1993	Low	Unclear	Low	Low	Low	Low	Low
De'Oliveira 1995	High	Low	Low	Low	Low	Low	Low
Jayne 1995	Low	Low	Low	Low	Low	Low	Low
Ara 1999	Low	Unclear	Low	Low	Low	Low	High
Segelmark 2003	High	Low	Low	Low	Unclear	Low	Low
Terrier 2009	Low	Low	Unclear	Low	Low	Low	Low
Miloslavsky 2013	Low	Low	Low	Low	Unclear	Low	Low
Specks 2013	High	Low	Low	Low	Unclear	Low	Low

Supplementary table S3. Summary of QUADAS-2 - Having detectable ANCA when a relapse is diagnosed. Green= Low risk of bias, Blue=unclear, Red=High risk of bias

Study	Risk of bias				Applicability concerns		
	Patient selection	Index test	Reference standard	Flow and timing	Patient selection	Index test	Reference standard
Jayne 1995	Low	Low	Low	Low	Low	Low	Low
Nowack 2001	Low	Low	Low	Low	Low	Low	Low
Han 2003	Low	Low	Low	Low	Low	Low	Low
Finkielman 2007	Low	Low	Low	Low	Low	Low	Low
Kemna 2014	Low	Low	Low	Low	Low	Low	Low
Fussner 2016	Low	Low	Low	Low	Low	Low	Low

Supplementary table S4. Summary of QUADAS-2 - Having a relapse when ANCA rises.
Green= Low risk of bias

Study	Risk of bias				Applicability concerns		
	Patient selection	Index test	Reference standard	Flow and timing	Patient selection	Index test	Reference standard
Dolman 1993	High	Low	Low	Low	Low	Low	Low
De'Oliveira 1995	High	Low	Low	Low	Low	Low	Low
Jayne 1995	Low	Low	Low	Low	Low	Low	Low
Boomsma 2000	Low	Low	Low	Low	Low	Low	Low
Nowack 2001	Low	Low	Low	Low	Low	Low	High
Han 2003	Low	Low	Low	High	Low	High	Low
Lurati-Ruiz 2005	Low	Low	Low	Low	Low	Low	High
Finkielman 2007	Low	Low	Low	Low	Low	Low	Low
Damoiseaux 2009	High	Low	Low	Low	Unclear	Low	Low
Terrier 2009	Low	High	Low	Low	Low	Low	Unclear
Kemna 2014	Low	Low	Low	Low	Low	Low	Low
Jones 2015	High	High	Low	Low	Low	Low	Low
Yamaguchi 2015	Low	Low	Low	High	Low	Low	High
Fussner 2016	Low	Low	Low	Low	Low	Low	Low
Watanabe 2018	Low	High	Low	Low	Low	Low	High
McClure 2019	Low	Low	Low	Low	Low	Low	Low

Supplementary table S5. Summary of QUADAS-2 - Having a relapse within 6 or 12 months after ANCA rise. Green= Low risk of bias, Blue=unclear, Red=High risk of bias

Study	Risk of bias	Applicability concerns	Details	↑ or ↓ estimation OR
Dolman 1993	Patient selection		Case-control study	↑
	Index test		Unclear cutoff value positive ANCA test	?
De'Oliveira 1995	Patient selection		Follow-up in selection of patients	?
Ara 1999	Index test		Unclear whether increase in titer was above cutoff value positive ANCA test	?
		Reference standard	Relapse defined as BVAS score >5	?
Segelmark 2003	Patient selection		Case-control study	↑
		Patient selection	Unclear study population	?
Terrier 2009	Reference standard		Unclear relapse definition (no BVAS score)	?
Miloslavsky 2013		Patient selection	Unclear study population	?
Specks 2013	Patient selection		Exclusion of very sick and relatively healthy patients	?
		Patient selection	Unclear study population	?

Supplementary table S6. Risk of bias details - Studies investigating detectable ANCA when a relapse is diagnosed.

Study	Risk of bias	Applicability concerns	Details	↑ or ↓ estimation OR
Dolman 1993	Patient selection		Case-control study	↑
De'Oliveira 1995	Patient selection		Follow-up in selection of patients	?
Nowack 2001		Reference standard	Inclusion of relapses simultaneous with ANCA rise	↑
Han 2003	Flow and Timing		Pre-emptive treatment in patients	↓
		Index test	Definition rise: 4-fold increase	?
Lurati-Ruiz 2005		Reference standard	Inclusion of relapses simultaneous with ANCA rise	↑
Damoiseaux 2009	Patient selection		Case-control study	↑
		Patient selection	Unclear study population	?
Terrier 2009	Index test		Rise defined as seroconversion	?
		Reference standard	Unclear time between ANCA rise and relapse	?
Jones 2015	Patient selection		Selection of patients who became ANCA-negative after induction	?
	Index test		Rise defined as seroconversion	?
Yamaguchi 2015	Flow and Timing		Pre-emptive treatment in patients	↓
		Reference standard	Inclusion of relapses simultaneous with ANCA rise	↑
Watanabe 2018	Index test		Rise defined as seroconversion	?
		Reference standard	Inclusion of relapses simultaneous with ANCA rise	↑

Supplementary table S7. Risk of bias details - Studies investigating relapses within 6 or 12 months after ANCA rise.

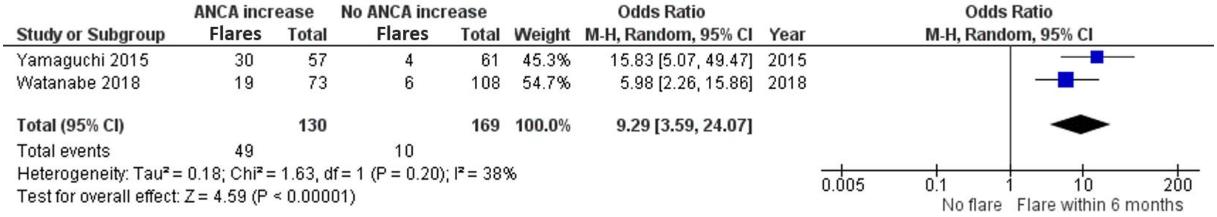

Supplementary figure S1. Meta-analysis summarizing the data regarding having a relapse within 6 months of a MPO-ANCA increase. Odds ratio with 95% confidence interval is displayed in the forest plot.

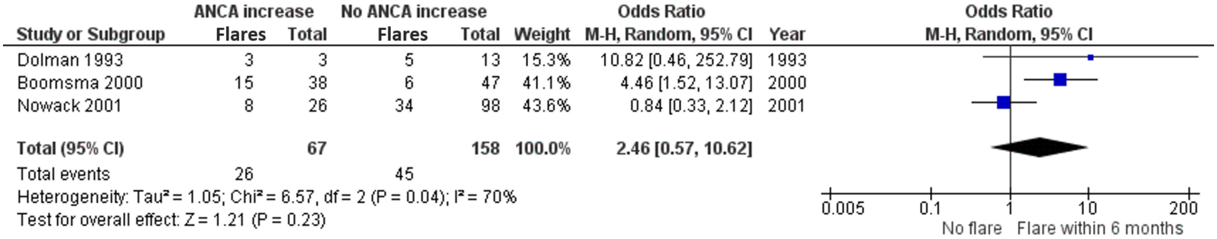

Supplementary figure S2. Meta-analysis summarizing the data regarding having a relapse within 6 months of a PR3-ANCA increase. Odds ratio with 95% confidence interval is displayed in the forest plot.

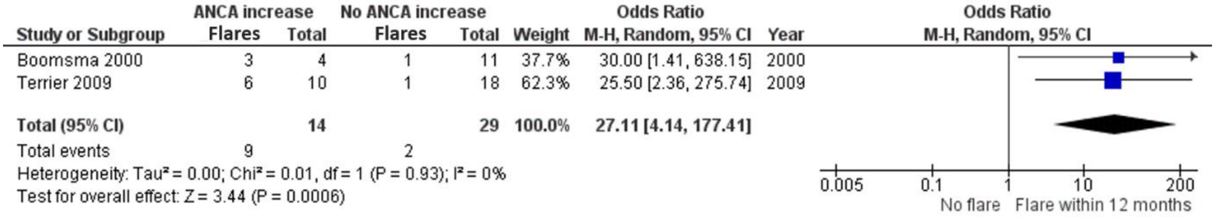

Supplementary figure S3. Meta-analysis summarizing the data regarding having a relapse within 12 months of a MPO-ANCA increase. Odds ratio with 95% confidence interval is displayed in the forest plot.

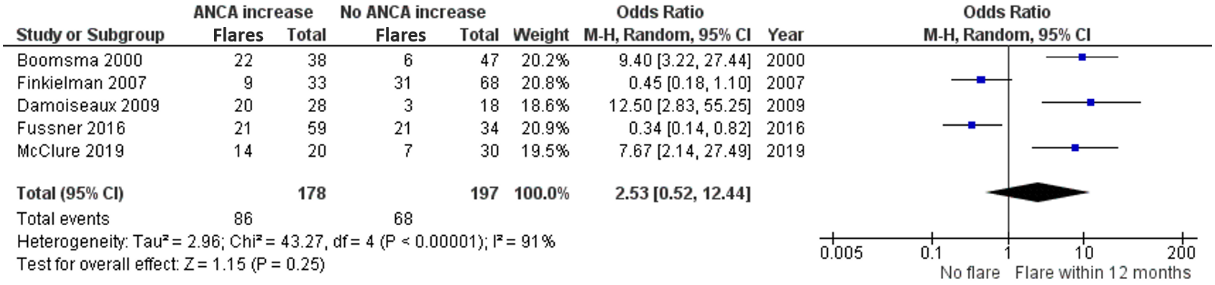

Supplementary figure S4. Meta-analysis summarizing the data regarding having a relapse within 12 months of a PR3-ANCA increase. Odds ratio with 95% confidence interval is displayed in the forest plot.
