# Supplementary figures and images for: Do Relapses Follow ANCA Rises? A Systematic Review and Meta-Analysis on the Value of Serial ANCA Level Evaluation
Source: Front Med (Lausanne). 2022 Jul 4;9:844112. doi: 10.3389/fmed.2022.844112 (PMC9289208; doi:10.3389/fmed.2022.844112)

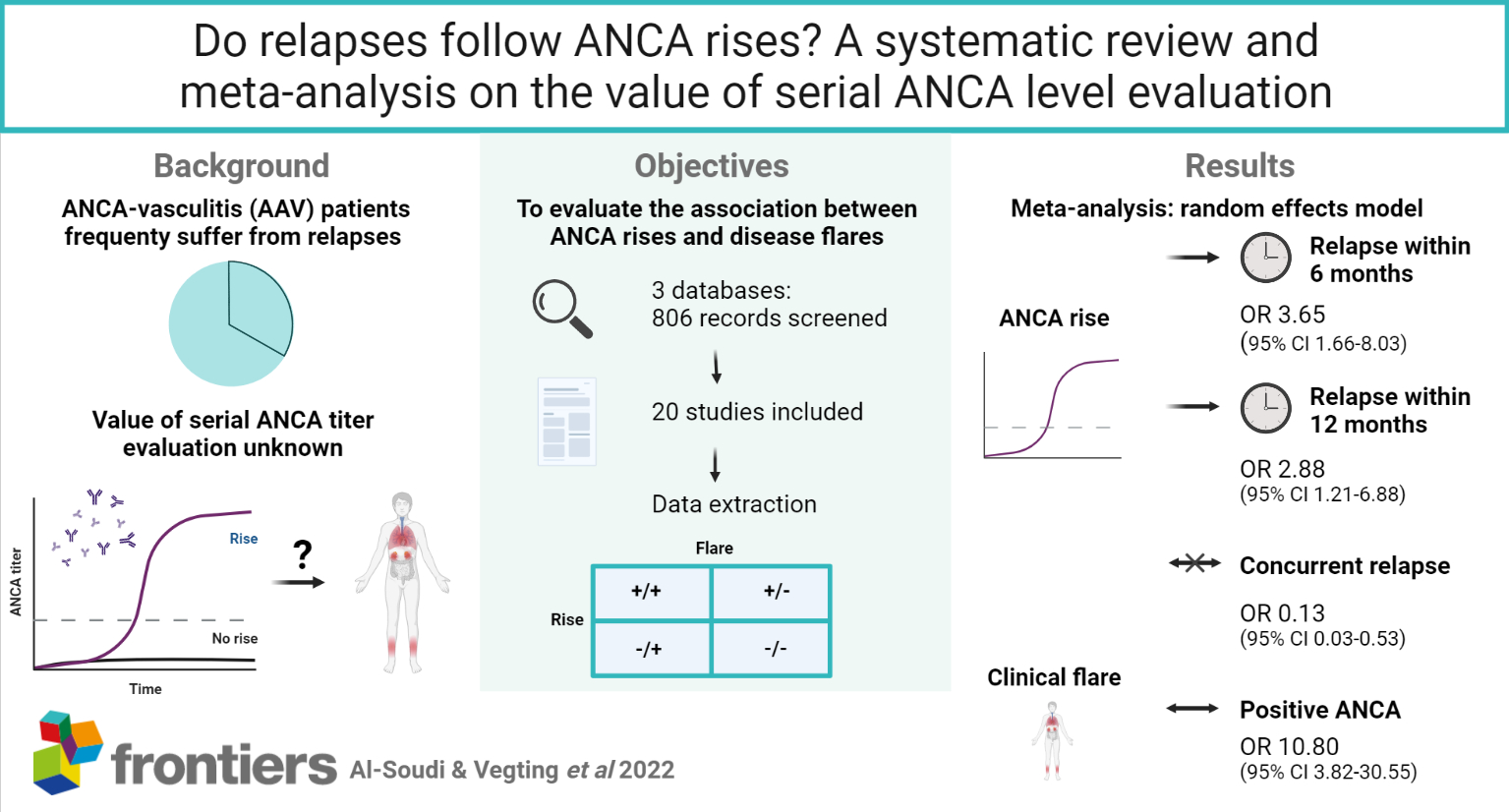

Supplement: Supplementary file 2 [file Image_1.jpeg]
